# Racial and ethnic differences in the utilization of autologous transplantation for lymphoma in the United States

**DOI:** 10.1002/cam4.4249

**Published:** 2021-09-01

**Authors:** John L. Vaughn, Orysya Soroka, Narendranath Epperla, Monika Safford, Laura C. Pinheiro

**Affiliations:** ^1^ Division of Hematology & Medical Oncology Weill Cornell Medicine New York New York USA; ^2^ Clinical Epidemiology & Health Services Research Program Weill Cornell Medicine New York New York USA; ^3^ Division of General Internal Medicine Weill Cornell Medicine New York New York USA; ^4^ Division of Hematology The Ohio State University Columbus Ohio USA

**Keywords:** autologous transplantation, healthcare disparities, hematopoietic stem cell transplantation, Hodgkin lymphoma, Non‐Hodgkin lymphoma

## Abstract

**Background:**

Racial/ethnic disparities in the utilization of hematopoietic cell transplantation (HCT) have been reported for patients with hematologic malignancies, but population‐based data are lacking for lymphoma patients. The objective of this study was to determine whether racial and ethnic disparities exist in the utilization of autologous HCT for lymphoma in the United States.

**Method:**

We used Surveillance, Epidemiology, and End Results data linked to Medicare fee‐for‐service claims. We included Medicare beneficiaries aged 66+ years with Hodgkin or Non‐Hodgkin lymphomas diagnosed between 2008 and 2015. The primary outcome was time‐to‐autologous HCT. We used Cox proportional hazards models to estimate racial/ethnic differences in utilization. Missing data were handled using multiple imputation with chained equations.

**Results:**

We included 40,605 individuals with lymphoma. A total of 452 autologous transplants were performed. In the unadjusted model, Non‐Hispanic Black patients were 51% less likely to receive a transplant than Non‐Hispanic White patients (95% CI, 0.26–0.96; *p *= 0.04). After adjusting for age at diagnosis and sex, Non‐Hispanic Black patients were 61% less likely to receive a transplant (95% CI, 0.20–0.76; *p *= 0.01). However, observed differences attenuated and became non‐significant after adjustment for socioeconomic factors (adjusted hazard ratio [aHR], 0.62; 95% CI, 0.32–1.21; *p* = 0.16) and disease‐specific factors (aHR, 0.58; 95% CI, 0.30–1.12; *p *= 0.11), separately. In the fully adjusted model, we also did not observe a statistically significant association between Non‐Hispanic Black race/ethnicity and receipt of transplant (aHR, 0.54; 95% CI, 0.28–1.05; *p *= 0.07).

**Conclusion:**

In this population‐based cohort study of lymphoma patients, Non‐Hispanic Black patients were less likely to receive autologous HCT compared to Non‐Hispanic White patients, but this difference was partially explained by socioeconomic and disease‐specific factors.

## Graphical abstract

Non‐Hispanic Black patients diagnosed with lymphoma between 2008 and 2015 were 51% less likely to receive an autologous transplant compared to Non‐Hispanic White patients. This difference was partially explained by socioeconomic and disease‐specific factors.

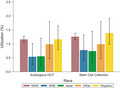

## 1 INTRODUCTION

In the United States, approximately 90,000 cases of lymphoma are diagnosed each year and 20,000 individuals die from their disease.^1^ Autologous hematopoietic cell transplantation (HCT) is considered the standard‐of‐care treatment for relapsed and refractory Hodgkin lymphoma (HL) and Non‐Hodgkin lymphoma (NHL) because it has been shown to improve outcomes including overall survival.^2^ According to data from the Center for International Blood and Marrow Transplant Research (CIBMTR), approximately 1000 patients with HL and 2500 patients with NHL receive this potentially life‐saving therapy each year.^3^ Collectively, lymphoma is the second most common indication for autologous HCT behind plasma cell disorders.^3^ Despite the importance of autologous HCT in the treatment of lymphoma, previous studies have shown that racial/ethnic minorities are less likely to receive this therapy compared to Non‐Hispanic White patients^4^, ^5^, ^6^, ^7^ However, data are lacking for lymphoma patients diagnosed since the early 2000s, and the factors that contribute to racial/ethnic differences in transplant utilization have been understudied for these patients.^8^

The objective of this study was to determine whether racial and ethnic disparities exist in the utilization of autologous HCT for lymphoma using the SEER‐Medicare linked database for patients diagnosed between 2008 and 2015. Since autologous HCT is a potentially life‐saving therapy for patients with lymphoma, it is important to understand whether disparities in treatment exist that might lead to inferior survival for minorities. In addition, it remains unclear whether demographic, socioeconomic, and disease‐specific factors account for lower autologous HCT utilization among racial/ethnic minorities. Importantly, since autologous HCT does not require the availability of a donor, differences in treatment utilization cannot be confounded by donor availability. Based on prior research, we hypothesized that Non‐Hispanic Black patients would underutilize autologous HCT even after adjustment for potential confounders.

## 2 METHOD

### 2.1 Data source

This was a population‐based cohort study using SEER cancer registry data (2008–2015) linked to Medicare fee‐for‐service claims (2007–2016). The version of SEER data used in this study was the SEER‐18 database, which covers 28% of the US cancer population.^9^ Medicare is a federally funded health insurance program that provides health insurance coverage to adults aged 65 years or older. Part A provides coverage for inpatient hospital care, skilled nursing facility care, some home healthcare, and hospice care. Part B provides coverage for physician visits, outpatient services, preventive services, and some home healthcare.^9^ Individuals included in the SEER database were linked to their Medicare claims every 2 years using personal identifiers. Approximately 96% of individuals aged 65 years or older in the SEER database are matched to Medicare claims.^9^ This study was determined exempt from review by the Institutional Review Board at Weill Cornell Medicine.

### 2.2 Cohort

We included individuals aged 66 years or older who were diagnosed with HL or NHL between 2008 and 2015. The age 66 years was selected so that eligible individuals would have 1 year of continuous Medicare fee‐for‐service claims in the year prior to their cancer diagnosis in order to calculate their Charlson Comorbidity Index score. SEER provides only the month and year of an individual's cancer diagnosis date. In order to identify cancer diagnosis date, we set the day of diagnosis to the 15th day of each month.^10^ Eligible individuals were required to have to continuous coverage in Medicare Parts A and B for 1 year prior to diagnosis to at least 3 months after diagnosis unless they died following diagnosis. We excluded individuals who were diagnosed by autopsy or death certificate, those who lacked microscopic confirmation of their lymphoma, and those enrolled in Medicare Part C (managed care) plans.

### 2.3 Study outcomes

The primary outcome was receipt of autologous HCT. This was identified using a combination of Current Procedural Terminology (CPT) and International Classification of Diseases (ICD) codes (Table S1). We operationalized this as a time‐to‐event variable. Time‐to‐stem cell collection was identified as an exploratory outcome. For both autologous HCT and stem cell collection, only incident outcomes were considered. We obtained CPT and ICD codes through a focused literature review, American Society of Transplantation and Cellular Therapy billing guidelines, discussion with billing physicians, and manual review of codes.^11^ We used a combination of inpatient and outpatient billing codes to avoid missing outcomes. We identified outcomes at any point following the diagnosis of lymphoma.

### 2.4 Key independent variable

The key independent variable was race/ethnicity. Race/ethnicity was defined based on the SEER race recode and origin variables.^12^ SEER reports race as White, Black, American Indian or Alaska Native, or Asian or Pacific Islander. SEER reports ethnicity as Hispanic or Non‐Hispanic. Hispanic ethnicity is determined using the North American Association of Central Cancer Registries algorithm.^13^ We combined the race and ethnicity variables into five categories: Non‐Hispanic White, Non‐Hispanic Black, Asian or Pacific Islander, Other, and Hispanic.^14^ The Other category included American Indians, Alaska Natives, and individuals with unknown race/ethnicity.

### 2.5 Covariates

We selected covariates based on Andersen's Behavioral Model of Health Services Use.^15^, ^16^ Covariates included predisposing factors (age at cancer diagnosis and biologic sex), enabling factors (marital status, Distressed Communities Index, region, distance to nearest transplant center, and year of cancer diagnosis), and need factors/illness level (Charlson Comorbidity Index, lymphoma subtype, and Ann Arbor stage). The Distressed Communities Index is a composite measure of socioeconomic status that combines seven complimentary socioeconomic metrics into a single score at the ZIP code level. These seven metrics encompass three separate socioeconomic status domains: education, income, and occupation. ZIP codes are then sorted into quintiles with higher scores corresponding to more distressed areas.^17^ Further description of the DCI metrics is provided in Table S2. The use of a composite variable for socioeconomic status avoids the problem of multicollinearity.^18^ We obtained a list of transplant centers from the National Marrow Donor Program.^19^ The driving distance to the nearest transplant center was calculated at the ZIP code level using the Microsoft Bing Maps Application Programming Interface. The patient ZIP code at the time of diagnosis was used. If the ZIP code was outside the continental United States, we used the Haversine distance instead. Charlson Comorbidity Index was calculated as previously described.^20^, ^21^, ^22^

### 2.6 Statistical analysis

We first compared differences in baseline characteristics using chi‐squared tests. We described receipt of autologous HCT and stem cell collections by calculating the proportion of patients receiving each service with exact 95% CIs. We modeled each outcome using Cox proportional hazards models.^23^ We censored patients at the time of death, loss of continuous enrollment in Medicare Parts A and B, or the end of the study (31 December 2016). We evaluated the proportional hazards assumption for race/ethnicity by assessing log–log plots and testing Schoenfeld residuals. We explored interactions between race/ethnicity and each covariate separately. For each outcome, we created five models with different specifications: (1) unadjusted, (2) adjusted for predisposing factors, (3) adjusted for enabling factors, (4) adjusted for need factors, and (5) fully adjusted. Age at diagnosis, distance to nearest transplant center, and year of diagnosis were modeled as continuous variables using restricted cubic splines with three knots.^24^ Adjusted hazard ratios (aHRs) with 95% CI were estimated for each race/ethnicity. We performed several subgroup analyses: patients with diffuse large B‐cell lymphoma (DLBCL) only; patients with DLBCL, T‐cell lymphomas (TCL), mantle cell lymphoma (MCL), and Hodgkin lymphoma (HL) only (i.e., excluding indolent lymphomas); and patients aged 66–74 years only (i.e., excluding the oldest patients in the cohort). Missing data were handled using multiple imputation with chained equations.^25^ Since the fraction of missing data was <10% for every variable, we used 10 imputations. Multinomial logistic regression was used for the imputation procedure, and the regression model included the study outcomes to avoid introducing bias into the results.^26^ Statistical tests were performed at a two‐sided α of 0.05. Statistical analyses were conducted in SAS version 9.4 and Stata version 16.1.

## 3 RESULTS

### 3.1 Cohort characteristics

**FIGURE 1:**
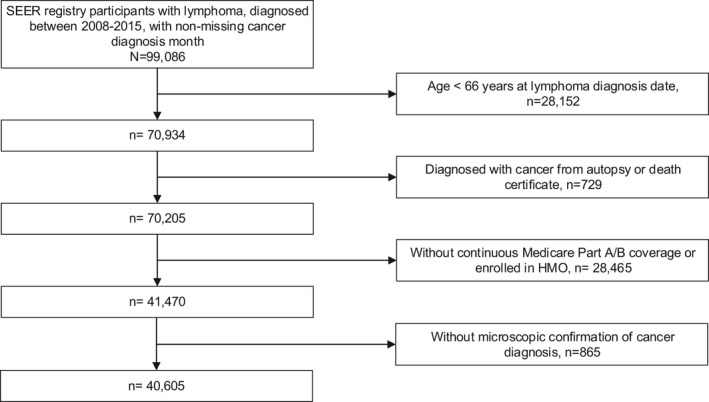
Flowchart for study population

We included 40,605 adults with lymphoma diagnosed between 2008 and 2015 (Figure 1). The most frequent type of lymphoma was DLBCL (37%) followed by follicular lymphoma (17%), marginal zone lymphomas (11%), T‐cell lymphomas (7%), mantle cell lymphoma (5%), Hodgkin lymphoma (4%), and other lymphomas (19%). Most patients were Non‐Hispanic White (84%) followed by Hispanic (7%), Non‐Hispanic Black (4%), Asian (3%), and Other (2%). Compared to Non‐Hispanic White patients, Non‐Hispanic Black patients were more likely to be younger at diagnosis (median age 74, vs. 77 years, *p *< 0.001), female (55% vs. 48%, *p* < 0.001), unmarried (14% vs. 6%, *p* < 0.001), reside within 60 miles of a National Marrow Donor Program transplant center (75% vs. 67%, *p* = 0.003), reside in an area with a DCI of 5 (41% vs. 12%, *p* < 0.001), reside in in the southern United States (41% vs. 24%, *p *< 0.001), and have two or more points on the Charlson Comorbidity Index (46% vs. 32%, *p *< 0.001). Patient characteristics for other races/ethnicities are shown in Table S3. Missing data were present for marital status (8%), Distressed Communities Index (3%), and Ann Arbor stage (7%).

### 3.2 Autologous HCT

**FIGURE 2:**
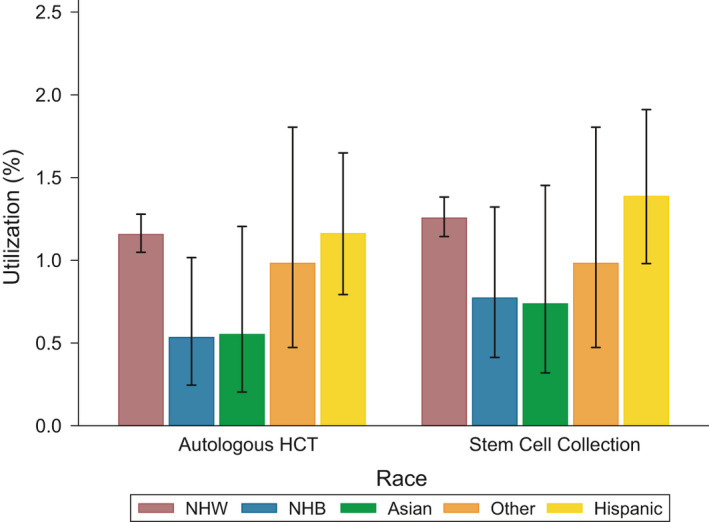
Autologous HCT and stem cell collections for Hodgkin and Non‐Hodgkin lymphomas. Utilization (%) refers to the proportion of lymphoma patients who received an autologous transplant or stem cell collection. Error bars refer to 95% exact confidence intervals for proportions

A total of 452 transplants were performed for an overall utilization rate of 1.1%. Median follow‐up was 2.4 years for Non‐Hispanic Whites (interquartile range [IQR], 0.9–4.7), 2.1 years for Non‐Hispanic Blacks (IQR, 0.6–4.3), 1.8 years for Asians (IQR, 0.5–4.1), and 2.0 years for Hispanics (IQR, 0.6–4.3). Autologous HCT utilization was highest for mantle cell lymphoma (4.4%) followed by T‐cell lymphomas (2.3%), DLBCL (1.3%), HL (1.2%), follicular lymphoma (0.5%), other lymphomas (0.4%), and marginal zone lymphomas (<1%). Crude transplant utilization rates for each race/ethnicity are shown in Figure 2. Transplant utilization was highest for Non‐Hispanic Whites and Hispanics (1.2% for both groups) followed by Asians (<1%) and Non‐Hispanic Blacks (<1%). Median time from diagnosis to transplant was 1.0 years (IQR, 0.6–1.5) for Non‐Hispanic Whites, 0.6 years (IQR, 0.5–2.0) for Non‐Hispanic Blacks, 0.6 years (IQR, 0.5–1.1) for Asians, and 1.1 years (IQR, 0.6–1.4) for Hispanics. Unadjusted and adjusted HRs for the association between race/ethnicity and autologous HCT utilization are shown in Table 1. In the unadjusted model, Non‐Hispanic Black patients were 51% less likely to receive a transplant than Non‐Hispanic White patients (95% CI, 0.26–0.96; *p* = 0.04). After adjusting for age at diagnosis and sex, Non‐Hispanic Black patients were 61% less likely to receive a transplant (95% CI, 0.20–0.76; *p *= 0.01). However, observed differences attenuated and became non‐significant after adjustment for socioeconomic factors (aHR, 0.62; 95% CI, 0.32–1.21; *p* = 0.16) and disease‐specific factors (aHR, 0.58; 95% CI, 0.30–1.12; *p* = 0.11), separately. In the fully adjusted model, we also did not observe a statistically significant association between Non‐Hispanic Black race/ethnicity and receipt of transplant (aHR, 0.54; 95% CI, 0.28–1.05; *p *= 0.07).

**TABLE 1:** Cox models for autologous HCT for Hodgkin and Non‐Hodgkin lymphomas

Race/ethnicity	Unadjusted		Adjusted for predisposing factors^a^		Adjusted for enabling factors^b^		Adjusted for need factors^c^		Fully adjusted	
	HR (95% CI)	*p* value	HR (95% CI)	*p* value	HR (95% CI)	*p* value	HR (95% CI)	*p* value	HR (95% CI)	*p* value
NHW	Reference		Reference		Reference		Reference		Reference	
NHB	0.49 (0.26–0.96)	0.04	0.39 (0.20–0.76)	0.01	0.62 (0.32–1.21)	0.16	0.58 (0.30–1.12)	0.11	0.54 (0.28–1.05)	0.07
Asian	0.55 (0.24–1.23)	0.14	0.63 (0.28–1.42)	0.26	0.60 (0.27–1.37)	0.23	0.52 (0.23–1.16)	0.11	0.61 (0.27–1.39)	0.24
Other	0.87 (0.46–1.62)	0.65	0.66 (0.35–1.23)	0.19	0.91 (0.48–1.73)	0.77	0.80 (0.43–1.50)	0.49	0.66 (0.35–1.24)	0.19
Hispanic	1.08 (0.75–1.56)	0.68	0.96 (0.66–1.38)	0.81	1.21 (0.83–1.76)	0.33	1.10 (0.76–1.59)	0.61	1.04 (0.71–1.52)	0.85

Abbreviations: CI, confidence interval; HCT, hematopoietic cell transplantation; HR, hazard ratio; NHB, Non‐Hispanic Black; NHW, Non‐Hispanic White.

^a^ Predisposing factors include age and sex.

^b^ Enabling factors include marital status, Distressed Communities Index, region, distance to nearest transplant center, and year of diagnosis.

^c^ Need factors include Charlson Comorbidity Index, lymphoma subtype, and Ann Arbor stage.

### 3.3 Stem cell collection

A total of 498 stem cell collections were performed for a utilization rate of 1.2%. As with autologous HCT, utilization was highest for mantle cell lymphoma (4.5%) followed by T‐cell lymphomas (2.4%), DLBCL (1.6%), HL (1.4%), follicular lymphoma (0.6%), other lymphomas (0.4%), and marginal zone lymphomas (<1%). Collection rates for each/ethnicity are shown in Figure 2. Median time from diagnosis to stem cell collection was 0.9 years (IQR, 0.5–1.4) for Non‐Hispanic Whites, 0.7 years (IQR, 0.5–1.8) for Non‐Hispanic Blacks, 1.2 years (IQR, 0.5–1.8) for Asians, and 0.9 years (IQR, 0.5–1.3) for Hispanics. Among patients who underwent stem cell collections, 83% of Non‐Hispanic Whites went on to receive transplants compared to 81% of Hispanics, 69% of Non‐Hispanic Blacks, and 63% of Asians. Unadjusted and adjusted HRs for the association between race/ethnicity and stem cell collections are shown in Table 2. In the unadjusted model, there were no significant racial/ethnic differences in utilization. The unadjusted HRs were 0.66 (95% CI, 0.38–1.14) for Non‐Hispanic Blacks, 0.67 (95% CI, 0.33–1.35) for Asians, and 1.18 (95% CI, 0.85–1.65) for Hispanics. In the age‐ and sex‐adjusted model, Non‐Hispanic Blacks were 48% less likely to undergo a stem cell collection procedure (95% CI, 0.30–0.90; *p *= 0.02). Similar to autologous HCT, the differences were attenuated and became non‐significant after adjustment for socioeconomic factors (aHR, 0.85; 95% CI, 0.481.48; *p *= 0.56) and disease‐specific factors (aHR, 0.77; 95% CI, 0.44–1.34; *p* = 0.35), separately. In the fully adjusted model, there was no statistically significant association between Non‐Hispanic Black race/ethnicity and receipt of stem cell collection (aHR, 0.72; 95% CI, 0.41–1.26; *p *= 0.25).

**TABLE 2:** Cox models for stem cell collections for Hodgkin and Non‐Hodgkin lymphomas

Race/ethnicity	Unadjusted		Adjusted for predisposing factors^a^		Adjusted for enabling factors^b^		Adjusted for need factors^c^		Fully adjusted	
	HR (95% CI)	*p* value	HR (95% CI)	*p* value	HR (95% CI)	*p* value	HR (95% CI)	*p* value	HR (95% CI)	*p* value
NHW	Reference		Reference		Reference		Reference		Reference	
NHB	0.66 (0.38–1.14)	0.13	0.52 (0.30–0.90)	0.02	0.85 (0.48–1.48)	0.56	0.77 (0.44–1.34)	0.35	0.72 (0.41–1.26)	0.25
Asian	0.67 (0.33–1.35)	0.26	0.77 (0.38–1.56)	0.47	0.69 (0.34–1.41)	0.31	0.62 (0.31–1.25)	0.18	0.68 (0.33–1.38)	0.29
Other	0.79 (0.42–1.49)	0.47	0.61 (0.32–1.14)	0.12	0.82 (0.43–1.55)	0.54	0.73 (0.39–1.37)	0.32	0.59 (0.31–1.10)	0.10
Hispanic	1.18 (0.85–1.65)	0.33	1.04 (0.74–1.45)	0.82	1.31 (0.92–1.85)	0.13	1.19 (0.85–1.67)	0.30	1.10 (0.77–1.56)	0.61

Abbreviations: CI, confidence interval; HR, hazard ratio; NHB, Non‐Hispanic Black; NHW, Non‐Hispanic White.

^a^ Predisposing factors include age and sex.

^b^ Enabling factors include marital status, Distressed Communities Index, region, distance to nearest transplant center, and year of diagnosis.

^c^ Need factors include Charlson Comorbidity Index, lymphoma subtype, and Ann Arbor stage.

### 3.4 Subgroup analyses

In our subgroup analysis of patients with DLBCL (the most frequent disease histology in our study), we observed a similar pattern of utilization with Non‐Hispanic White patients having the highest utilization of both autologous HCT and stem cell collections (Figure S1 and Tables S4 and S5). However, given the reduced sample size and smaller number of transplants compared to the entire cohort, the differences were not statistically significant. The results were similar for our subgroup analysis of patients with DLBCL, TCL, MCL, and HL. These results are shown in Figure S2 and Tables S6 and S7. Finally, in our subgroup analysis of patients aged 66–74 years, we found that Non‐Hispanic Black patients were 59% less likely to receive a transplant compared to Non‐Hispanic White patients in the unadjusted model (95% CI, 0.21–0.80; *p *= 0.01). Non‐Hispanic Black patients were also 45% less likely to receive a stem cell collection in the unadjusted model (95% CI, 0.32–0.96; *p *= 0.04). However, these differences attenuated and became non‐significant in the fully adjusted models (Figure S3 and Tables S8 and S9. Interestingly, in the fully adjusted model for stem cell collections including only patients aged 66–74 years, we found that patients with unspecified race/ethnicity (including American Indians, Alaska Natives, and those with unknown/race ethnicity) were 56% less likely to receive a collection compared to Non‐Hispanic White patients, though this finding was considered exploratory (95% CI, 0.21–0.94; *p* = 0.03) (Table S9).

## 4 DISCUSSION

In this population‐based cohort study of adults with lymphoma diagnosed in the United States between 2008 and 2015, we found that autologous HCT utilization was highest for Non‐Hispanic Whites and Hispanics followed by Asians and Non‐Hispanic Blacks. However, differences in autologous HCT utilization by race/ethnicity were only statistically significant between Non‐Hispanic Black patients compared to Non‐Hispanic White patients. This observed difference in utilization persisted after adjustment for age and sex but was no longer statistically significant after adjustment for socioeconomic and disease‐specific factors. Non‐Hispanic Black patients were more than three times more likely to reside in the most economically distressed areas (defined as a DCI score of 5), and they had a higher comorbidity burden at the time of diagnosis. This suggests that racial/ethnic differences in autologous HCT utilization for lymphoma are partially explained by differences in these factors.

One of the earliest studies to investigate racial/ethnic differences in HCT utilization for lymphoma was published by Mitchell et al. in 1997.^5^ The authors used inpatient hospital discharge data from California, Maryland, Massachusetts, and New York between 1988 and 1991. A total of 23,304 adults with HL and NHL were included. The proportion of patients receiving any type of HCT was 2.2%–3.4% depending on the state. After adjustment for age, sex, insurance type, year of diagnosis, and lymphoma subtype (HL or NHL), the adjusted odds ratio (aOR) of receiving any type of HCT for lymphoma was 0.34–0.45 for Non‐Hispanic Blacks, 0.40–0.74 for Hispanics, 1.32 for Asians, and 0.60–2.60 for other minorities compared to Non‐Hispanic Whites. These differences were statistically significant for Non‐Hispanic Blacks in all four states, Hispanics in New York, and other racial minorities in Maryland/Massachusetts.

In a more recent study, Joshua et al. included 9482 patients with NHL diagnosed in the United States between 1997 and 2002.^4^ The authors estimated age‐adjusted ORs of receiving autologous HCT using aggregate data from the CIBMTR and Surveillance, Epidemiology, and End Results (SEER) database. Using Black race as the reference, the OR of receiving autologous HCT for NHL was 2.03 for Whites (95% confidence interval [CI], 1.86–2.22; *p *< 0.001]. Another study of California inpatient hospital discharge data included 9137 patients with lymphoma hospitalized between 2002 and 2003 and followed through 2005. The authors found that there was no significant difference in HCT utilization between racial groups after adjustment for age, sex, lymphoma subtype (HL or NHL), distance to treatment, insurance type, and socioeconomic indicators.^7^ Our study augments the existing literature by including a large population‐based cohort of patients diagnosed in the contemporary time period, adjusting for novel covariates such as the Distressed Communities Index and driving distance to nearest NMDP transplant center, and using multiple models to identify factors that contribute to racial/ethnic differences in utilization.

Although prior studies have reported racial/ethnic differences in HCT utilization for patients with hematologic malignancies, the factors contributing to these differences have been understudied. In a CIBMTR analysis of 28,450 patients with multiple myeloma, Non‐Hispanic Black patients had lower Karnofsky performance status and higher HCT‐Comorbidity Index prior to transplant compared to Non‐Hispanic White and Hispanic patients.^27^ In a single‐center study of 562 patients with multiple myeloma, Black patients were more likely to reside in lower median household income tertiles than White patients and were more likely to be uninsured at the time of diagnosis.^28^ However, differences in autologous HCT utilization persisted even after adjustment for these factors.^29^ In a single‐center study of patients with acute myeloid leukemia, the authors manually reviewed charts to determine the reasons why allogeneic HCT was not performed for Black patients with intermediate‐ or high‐risk karyotypes.^30^ They found that documented reasons included older age (27%), refractory disease (21%), comorbidities or poor performance status (16%), death (9%), lack of available donor (6%), drug use or incarceration (6%), and offered but declined (3%). Given differences between allogeneic and autologous HCT, it is likely that the frequency of those reasons would differ for lymphoma patients being evaluated for autologous HCT. Additional research is needed to understand barriers to transplantation from the perspective of patients using mixed methods. Studies are also needed to understand the financial impact of transplantation on racial/ethnic minorities since socioeconomic status partially accounts for decreased utilization for Non‐Hispanic Black patients.

### 4.1 Limitations

Our cohort was limited to patients aged 66 years or older at the time of diagnosis. In the United States, the median age at diagnosis for NHL is 67 years.^31^ However, since younger patients are more likely to be eligible for autologous HCT, our cohort may not be representative of the patients who are most likely to receive a transplant. The small number of transplants within specific disease subgroups prevented us from performing subgroup analyses for most lymphoma subtypes. However, we observed a similar pattern of utilization in several different subgroups. We also adjusted for disease histology in our models to account for different distributions of aggressive and indolent lymphomas between racial/ethnic groups. We were unable to determine the number of prior lines of therapy prior to transplant since complete treatment information (including Medicare Part D data) was not available for most patients in the cohort. Additional limitations include exclusion of some states with high‐volume transplant centers (e.g., Texas), exclusion of Medicare Part C beneficiaries, the small number of racial/ethnic minorities compared to Non‐Hispanic Whites, and the limited follow‐up for patients diagnosed at the end of the study period. Finally, our study was unable to account for unmeasured confounders including disease status at transplant, discrimination, and mistrust.^32^

## 5 CONCLUSION

In conclusion, we found that Non‐Hispanic Black patients were less likely to receive autologous HCT compared to Non‐Hispanic White patients. This difference in utilization was partially explained by socioeconomic and disease‐specific factors. In particular, Non‐Hispanic Black patients were more than three times more likely to reside in the most economically distressed areas, and they had a higher comorbidity burden at the time of diagnosis. After adjustment for these factors, no significant differences in utilization were observed. Our findings suggest that interventions to improve access to autologous transplantation should focus on reducing financial toxicity of transplantation and developing less toxic conditioning regimens for patients with comorbidities. With the advent of chimeric antigen receptor T‐cell therapy, the field of oncology is rapidly changing. In the future, fewer lymphoma patients may be offered autologous HCT. However, as lymphoma treatments become increasingly sophisticated and more patients are offered novel therapies, it will be critical to ensure equal access for disadvantaged populations.

## CONFLICT OF INTEREST

The authors declare no conflict of interest.

## Supporting information

Supplementary Material

## Data Availability

The data that support the findings of this study are available from SEER‐Medicare. Restrictions apply to the availability of these data, which were used under license for this study. Data are available at https://healthcaredelivery.cancer.gov/seermedicare/ with the permission of SEER‐Medicare.
