# Supplementary material for: Racial and ethnic differences in the utilization of autologous transplantation for lymphoma in the United States
Source: Cancer Med. 2021 Sep 1;10(20):7330–8. doi: 10.1002/cam4.4249 (PMC8525101; doi:10.1002/cam4.4249)
Supplement: Supplementary file 1 — Supplementary Material [file CAM4-10-7330-s001.docx]

**Supplementary Appendix**

**Supplementary Table 1.** Codes used to identify autologous HCT and stem cell collections

**Supplementary Table 2.** Distressed Communities Index

**Supplementary Table 3.** Patient characteristics

**Supplementary Figure 1.** Autologous HCT and stem cell collections for DLBCL

**Supplementary Table 4.** Cox models for autologous HCT for DLBCL

**Supplementary Table 5.** Cox models for stem cell collections for DLBCL

**Supplementary Figure 2.** Autologous HCT and stem cell collections for DLBCL, TCL, MCL and HL

**Supplementary Table 6**. Cox models for autologous HCT for DLBCL, TCL, MCL and HL

**Supplementary Table 7**. Cox models for stem collections for HCT for DLBCL, TCL, MCL and HL

**Supplementary Figure 3.** Autologous HCT and stem cell collections for patients aged 66-74 years

**Supplementary Table 8**. Cox models for autologous HCT for patients aged 66-74 years

**Supplementary Table 9**. Cox models for stem collections for patients aged 66-74 years

**Supplementary Table 1.** Codes used to identify autologous HCT and stem cell collections

| **Autologous HCT** | | **Stem Cell Collection** |
| --- | --- | --- |
| CPT Codes | 38241 | 38206  38232 |
| ICD-9 Codes | 41.01  41.04  41.07  41.09 | None |
| ICD-10 Codes | 302.30G0  302.30X0  302.30Y0  302.33G0  302.33X0  302.33Y0  302.40G0  302.40X0  302.40Y0  302.43G0  302.43X0  302.43Y0  302.50G0  302.50X0  302.50Y0  302.53G0  302.53X0  302.53Y0  302.60G0  302.60X0  302.60Y0  302.63G0  302.63X0  302.63Y0  3E03005  3E03305  3E04005  3E04305  3E05005  3E05305  3E06005  3E06305  302.33AZ  302.40AZ  302.43AZ | None |

Abbreviations: CPT = Current Procedural Terminology, HCT = Hematopoietic Cell Transplantation, ICD = International Classification of Diseases

**Supplementary Table 2**. Distressed Communities Index

| **Metric** | **Definition** |
| --- | --- |
| No high school diploma | The share of the population age 25 and older who lack a high school diploma or equivalent. |
| Poverty rate | The share of individuals living below the federal poverty line. |
| Adults not working | The share of the population age 25 to 54 not working (either unemployed or not in the labor force). |
| Housing vacancy rate | The share of housing units that are vacant, adjusted for recreational, seasonal, or occasional use vacancies. |
| Median household income | Median household income as a percent of metro area or state median household income. |
| Change in employment | The change from 2014 to 2018 in the number of employees working in the geography. |
| Change in establishments | The change from 2014 to 2018 in the number of establishments located in the geography. |

**Supplementary Table 3.** Patient characteristics

|  | **NHW** | **NHB** | **Asian** | **Other** | **Hispanic** | ***P*-value** |
| --- | --- | --- | --- | --- | --- | --- |
| **Patients (n)** | 34,170 | 1,677 | 1,081 | 1,015 | 2,662 |  |
| **Age Group** |  |  |  |  |  | <0.001 |
| 66-69 | 5,506 (16%) | 398 (24%) | 126 (12%) | 235 (23%) | 530 (20%) |  |
| 70-74 | 7,605 (22%) | 447 (27%) | 227 (21%) | 246 (24%) | 638 (24%) |  |
| 75-79 | 7,680 (22%) | 352 (21%) | 233 (22%) | 267 (26%) | 619 (23%) |  |
| 80-84 | 6,989 (20%) | 271 (16%) | 241 (22%) | 155 (15%) | 485 (18%) |  |
| 85+ | 6,390 (19%) | 209 (12%) | 254 (23%) | 112 (11%) | 390 (15%) |  |
| **Sex** |  |  |  |  |  | <0.001 |
| Male | 17,651 (52%) | 756 (45%) | 546 (51%) | 567 (56%) | 1,282 (48%) |  |
| Female | 16,519 (48%) | 921 (55%) | 535 (49%) | 448 (44%) | 1,380 (52%) |  |
| **Marital Status** |  |  |  |  |  | <0.001 |
| Single | 2,156 (6%) | 235 (14%) | 72 (7%) | 85 (8%) | 262 (10%) |  |
| Married | 18,661 (55%) | 634 (38%) | 604 (56%) | 596 (59%) | 1,306 (49%) |  |
| Other | 10,498 (31%) | 665 (40%) | 320 (30%) | 247 (24%) | 893 (34%) |  |
| Missing | 2,855 (8%) | 143 (9%) | 85 (8%) | 87 (9%) | 201 (8%) |  |
| **Distance to Nearest Transplant Center** |  |  |  |  |  | <0.001 |
| <=60 Miles | 22,808 (67%) | 1,255 (75%) | 903 (84%) | 663 (65%) | 1,785 (67%) |  |
| >60-180 Miles | 9,943 (29%) | 384 (23%) | * | 137 (13%) | 556 (21%) |  |
| >180-360 Miles | 1,182 (3%) | * | * | 35 (3%) | 284 (11%) |  |
| >360 Miles | 237 (1%) | * | 127 (12%) | 180 (18%) | 37 (1%) |  |
| **Geographic Region** |  |  |  |  |  | <0.001 |
| West | 13,372 (39%) | 388 (23%) | 956 (88%) | 815 (80%) | 2,063 (77%) |  |
| Northeast | 7,875 (23%) | 331 (20%) | 86 (8%) | 103 (10%) | 445 (17%) |  |
| Midwest | 4,648 (14%) | 264 (16%) | 22 (2%) | 57 (6%) | 57 (2%) |  |
| South | 8,275 (24%) | 694 (41%) | 17 (2%) | 40 (4%) | 97 (4%) |  |
| **Distressed Communities Index** |  |  |  |  |  | <0.001 |
| 1 (Least Distressed) | 10,391 (30%) | 157 (9%) | 294 (27%) | 314 (31%) | 422 (16%) |  |
| 2 | 8,735 (26%) | 244 (15%) | 309 (29%) | 285 (28%) | 481 (18%) |  |
| 3 | 5,819 (17%) | 220 (13%) | 238 (22%) | 170 (17%) | 578 (22%) |  |
| 4 | 4,617 (14%) | 314 (19%) | 163 (15%) | 159 (16%) | 648 (24%) |  |
| 5 (Most Distressed) | 3,677 (11%) | 680 (41%) | 65 (6%) | 53 (5%) | 428 (16%) |  |
| Missing | 931 (3%) | 62 (4%) | 12 (1%) | 34 (3%) | 105 (4%) |  |
| **Charlson Comorbidity Index** |  |  |  |  |  | <0.001 |
| 0 | 14,997 (44%) | 543 (32%) | 394 (36%) | 446 (44%) | 970 (36%) |  |
| 1 | 8,335 (24%) | 362 (22%) | 289 (27%) | 240 (24%) | 692 (26%) |  |
| 2+ | 10,838 (32%) | 772 (46%) | 398 (37%) | 329 (32%) | 1,000 (38%) |  |
| **Lymphoma Subtype** |  |  |  |  |  | <0.001 |
| DLBCL | 12,425 (36%) | 503 (30%) | 536 (50%) | 433 (43%) | 1,085 (41%) |  |
| Follicular Lymphoma | 5,933 (17%) | 215 (13%) | 102 (9%) | 155 (15%) | 431 (16%) |  |
| Marginal Zone Lymphomas | 3,829 (11%) | 237 (14%) | 123 (11%) | 109 (11%) | 304 (11%) |  |
| T-Cell Lymphomas | 2,222 (7%) | 208 (12%) | 90 (8%) | 77 (8%) | 166 (6%) |  |
| Mantle Cell Lymphoma | 1,828 (5%) | 56 (3%) | 32 (3%) | 34 (3%) | 130 (5%) |  |
| Hodgkin Lymphoma | 1,405 (4%) | 87 (5%) | 36 (3%) | 33 (3%) | 143 (5%) |  |
| Other Lymphomas | 6,528 (19%) | 371 (22%) | 162 (15%) | 174 (17%) | 403 (15%) |  |
| **Ann Arbor Stage** |  |  |  |  |  | <0.001 |
| Stage I-II | 13,612 (40%) | 636 (38%) | 442 (41%) | 399 (39%) | 1,049 (39%) |  |
| Stage III-IV | 17,083 (50%) | 835 (50%) | 501 (46%) | 505 (50%) | 1,356 (51%) |  |
| Not Applicable | 1,073 (3%) | 94 (6%) | 30 (3%) | 29 (3%) | 57 (2%) |  |
| Missing | 2,402 (7%) | 112 (7%) | 108 (10%) | 82 (8%) | 200 (8%) |  |
| **Period of Diagnosis** |  |  |  |  |  | <0.001 |
| 2008-2009 | 9,169 (27%) | 443 (26%) | 279 (26%) | 213 (21%) | 676 (25%) |  |
| 2010-2011 | 8,303 (24%) | 392 (23%) | 278 (26%) | 216 (21%) | 677 (25%) |  |
| 2012-2013 | 8,254 (24%) | 432 (26%) | 283 (26%) | 273 (27%) | 649 (24%) |  |
| 2014-2015 | 8,444 (25%) | 410 (24%) | 241 (22%) | 313 (31%) | 660 (25%) |  |

Abbreviations: CI = Confidence Interval, DLBCL = Diffuse Large B-cell Lymphoma, Non-Hispanic Black, NHW = Non-Hispanic White

Data are shown as n(%) unless otherwise specified.

*Data are not shown to protect patient confidentiality.

**Supplementary Figure 1.** Autologous HCT and stem cell collections for DLBCL

**Supplementary Table 4.** Cox models for autologous HCT utilization for DLBCL

| **Race/**  **Ethnicity** | **Unadjusted** | | **Adjusted for**  **Predisposing Factors*** | | **Adjusted for**  **Enabling Factors**** | | **Adjusted for**  **Need Factors***** | | **Fully Adjusted** | |
| --- | --- | --- | --- | --- | --- | --- | --- | --- | --- | --- |
|  | HR (95% CI) | *P*-value | HR (95% CI) | *P*-value | HR (95% CI) | *P*-value | HR (95% CI) | *P*-value | HR (95% CI) | *P*-value |
| NHW | Reference |  | Reference |  | Reference |  | Reference |  | Reference |  |
| NHB | 0.44 (0.14-1.39) | 0.16 | 0.35 (0.11-1.09) | 0.07 | 0.61 (0.19-1.94) | 0.41 | 0.49 (0.16-1.52) | 0.22 | 0.48 (0.15-1.53) | 0.22 |
| Asian | 0.59 (0.22-1.59) | 0.30 | 0.66 (0.25-1.78) | 0.41 | 0.60 (0.22-1.64) | 0.32 | 0.63 (0.23-1.69) | 0.36 | 0.67 (0.24-1.83) | 0.43 |
| Other | 0.77 (0.32-1.88) | 0.57 | 0.68 (0.28-1.66) | 0.40 | 0.77 (0.31-1.92) | 0.58 | 0.77 (0.31-1.86) | 0.56 | 0.60 (0.24-1.47) | 0.26 |
| Hispanic | 0.87 (0.50-1.53) | 0.63 | 0.74 (0.42-1.31) | 0.31 | 0.96 (0.54-1.72) | 0.90 | 0.93 (0.53-1.63) | 0.80 | 0.83 (0.46-1.48) | 0.53 |

Abbreviations: CI = Confidence Interval, DLBCL = diffuse large-B cell lymphoma, HCT = Hematopoietic Cell Transplantation, HR = Hazard Ratio, NHB = Non-Hispanic Black, NHW = Non-Hispanic White

*Predisposing factors include age and sex.

**Enabling factors include marital status, Distressed Communities Index, region, distance to nearest transplant center, and year of diagnosis.

***Need factors include Charlson Comorbidity Index and Ann Arbor stage.

**Supplementary Table 5.** Cox models for stem cell collections for DLBCL

| **Race/**  **Ethnicity** | **Unadjusted** | | **Adjusted for**  **Predisposing Factors*** | | **Adjusted for**  **Enabling Factors**** | | **Adjusted for**  **Need Factors***** | | **Fully Adjusted** | |
| --- | --- | --- | --- | --- | --- | --- | --- | --- | --- | --- |
|  | HR (95% CI) | *P*-value | HR (95% CI) | *P*-value | HR (95% CI) | *P*-value | HR (95% CI) | *P*-value | HR (95% CI) | *P*-value |
| NHW | Reference |  | Reference |  | Reference |  | Reference |  | Reference |  |
| NHB | 0.64 (0.26-1.56) | 0.33 | 0.50 (0.21-1.22) | 0.13 | 0.90 (0.37-2.21) | 0.82 | 0.69 (0.29-1.68) | 0.42 | 0.68 (0.28-1.66) | 0.39 |
| Asian | 0.89 (0.42-1.90) | 0.77 | 1.00 (0.47-2.12) | 0.99 | 0.88 (0.41-1.90) | 0.74 | 0.95 (0.45-2.02) | 0.89 | 0.98 (0.45-2.12) | 0.95 |
| Other | 0.80 (0.36-1.81) | 0.60 | 0.71 (0.32-1.61) | 0.42 | 0.81 (0.35-1.86) | 0.62 | 0.79 (0.35-1.79) | 0.58 | 0.63 (0.28-1.42) | 0.26 |
| Hispanic | 0.81 (0.47-1.39) | 0.45 | 0.69 (0.40-1.18) | 0.17 | 0.90 (0.51-1.57) | 0.71 | 0.86 (0.50-1.48) | 0.59 | 0.75 (0.43-1.31) | 0.31 |

Abbreviations: CI = Confidence Interval, DLBCL = Diffuse Large-B cell Lymphoma, HR = Hazard Ratio, NHB = Non-Hispanic Black, NHW = Non-Hispanic White

*Predisposing factors include age and sex.

**Enabling factors include marital status, Distressed Communities Index, region, distance to nearest transplant center, and year of diagnosis.

***Need factors include Charlson Comorbidity Index, and Ann Arbor stage.

**Supplementary Figure 2.** Autologous HCT and stem cell collections for DLBCL, TCL, MCL and HL

**Supplementary Table 6.** Cox models for autologous HCT for DLBCL, TCL, MCL and HL

| **Race/**  **Ethnicity** | **Unadjusted** | | **Adjusted for**  **Predisposing Factors*** | | **Adjusted for**  **Enabling Factors**** | | **Adjusted for**  **Need Factors***** | | **Fully Adjusted** | |
| --- | --- | --- | --- | --- | --- | --- | --- | --- | --- | --- |
|  | HR (95% CI) | *P*-value | HR (95% CI) | *P*-value | HR (95% CI) | *P*-value | HR (95% CI) | *P*-value | HR (95% CI) | *P*-value |
| NHW | Reference |  | Reference |  | Reference |  | Reference |  | Reference |  |
| NHB | 0.62 (0.32-1.20) | 0.15 | 0.48 (0.25-0.92) | 0.03 | 0.81 (0.41-1.58) | 0.53 | 0.72 (0.37-1.39) | 0.33 | 0.67 (0.34-1.31) | 0.24 |
| Asian | 0.54 (0.24-1.20) | 0.13 | 0.63 (0.28-1.41) | 0.26 | 0.57 (0.25-1.30) | 0.18 | 0.57 (0.26-1.29) | 0.18 | 0.68 (0.30-1.54) | 0.35 |
| Other | 0.93 (0.50-1.75) | 0.83 | 0.81 (0.43-1.53) | 0.52 | 0.96 (0.50-1.84) | 0.90 | 0.91 (0.48-1.70) | 0.76 | 0.75 (0.39-1.41) | 0.37 |
| Hispanic | 0.92 (0.60-1.39) | 0.68 | 0.81 (0.54-1.23) | 0.32 | 1.02 (0.66-1.56) | 0.94 | 0.97 (0.64-1.46) | 0.87 | 0.93 (0.60-1.43) | 0.74 |

Abbreviations: CI = Confidence Interval, DLBCL = Diffuse Large-B cell Lymphoma, HCT = Hematopoietic Cell Transplantation, HL = Hodgkin Lymphoma, HR = Hazard Ratio, MCL = Mantle Cell Lymphoma, NHB = Non-Hispanic Black, NHW = Non-Hispanic White, TCL = T-Cell Lymphomas

*Predisposing factors include age and sex.

**Enabling factors include marital status, Distressed Communities Index, region, distance to nearest transplant center, and year of diagnosis.

***Need factors include Charlson Comorbidity Index, lymphoma subtype, and Ann Arbor stage.

**Supplementary Table 7.** Cox models for stem collections for DLBCL, TCL, MCL and HL

| **Race/**  **Ethnicity** | **Unadjusted** | | **Adjusted for**  **Predisposing Factors*** | | **Adjusted for**  **Enabling Factors**** | | **Adjusted for**  **Need Factors***** | | **Fully Adjusted** | |
| --- | --- | --- | --- | --- | --- | --- | --- | --- | --- | --- |
|  | HR (95% CI) | *P*-value | HR (95% CI) | *P*-value | HR (95% CI) | *P*-value | HR (95% CI) | *P*-value | HR (95% CI) | *P*-value |
| NHW | Reference |  | Reference |  | Reference |  | Reference |  | Reference |  |
| NHB | 0.75 (0.42-1.33) | 0.32 | 0.58 (0.32-1.02) | 0.06 | 1.00 (0.56-1.79) | 1.00 | 0.86 (0.49-1.53) | 0.62 | 0.81 (0.45-1.45) | 0.48 |
| Asian | 0.65 (0.32-1.30) | 0.22 | 0.75 (0.37-1.52) | 0.43 | 0.64 (0.32-1.31) | 0.23 | 0.69 (0.34-1.39) | 0.30 | 0.75 (0.37-1.53) | 0.43 |
| Other | 0.85 (0.45-1.59) | 0.61 | 0.74 (0.39-1.38) | 0.34 | 0.86 (0.45-1.63) | 0.64 | 0.82 (0.44-1.54) | 0.55 | 0.67 (0.35-1.27) | 0.22 |
| Hispanic | 0.97 (0.66-1.42) | 0.87 | 0.85 (0.58-1.25) | 0.41 | 1.08 (0.72-1.60) | 0.71 | 1.02 (0.69-1.50) | 0.92 | 0.96 (0.64-1.42) | 0.82 |

Abbreviations: CI = Confidence Interval, DLBCL = Diffuse Large-B cell Lymphoma, HL = Hodgkin Lymphoma, HR = Hazard Ratio, MCL = Mantle Cell Lymphoma, NHB = Non-Hispanic Black, NHW = Non-Hispanic White, TCL = T-Cell Lymphomas

*Predisposing factors include age and sex.

**Enabling factors include marital status, Distressed Communities Index, region, distance to nearest transplant center, and year of diagnosis.

***Need factors include Charlson Comorbidity Index, lymphoma subtype, and Ann Arbor stage.

**Supplementary Figure 3.** Autologous HCT and stem cell collections for patients aged 66-74 years

**Supplementary Table 8.** Cox models for autologous HCT for patients aged 66-74 years

| **Race/**  **Ethnicity** | **Unadjusted** | | **Adjusted for**  **Predisposing Factors*** | | **Adjusted for**  **Enabling Factors**** | | **Adjusted for**  **Need Factors***** | | **Fully Adjusted** | |
| --- | --- | --- | --- | --- | --- | --- | --- | --- | --- | --- |
|  | HR (95% CI) | *P*-value | HR (95% CI) | *P*-value | HR (95% CI) | *P*-value | HR (95% CI) | *P*-value | HR (95% CI) | *P*-value |
| NHW | Reference |  | Reference |  | Reference |  | Reference |  | Reference |  |
| NHB | 0.41 (0.21-0.80) | 0.01 | 0.41 (0.21-0.79) | 0.01 | 0.52 (0.26-1.01) | 0.05 | 0.48 (0.25-0.94) | 0.03 | 0.56 (0.29-1.10) | 0.09 |
| Asian | 0.65 (0.29-1.46) | 0.30 | 0.67 (0.30-1.51) | 0.34 | 0.68 (0.30-1.54) | 0.36 | 0.59 (0.27-1.33) | 0.21 | 0.65 (0.29-1.48) | 0.31 |
| Other | 0.62 (0.31-1.24) | 0.18 | 0.55 (0.27-1.11) | 0.09 | 0.61 (0.30-1.25) | 0.18 | 0.58 (0.29-1.18) | 0.13 | 0.55 (0.27-1.13) | 0.10 |
| Hispanic | 0.98 (0.68-1.42) | 0.93 | 0.97 (0.67-1.40) | 0.86 | 1.08 (0.73-1.58) | 0.71 | 1.00 (0.69-1.45) | 0.99 | 1.05 (0.71-1.55) | 0.80 |

Abbreviations: CI = Confidence Interval, HCT = Hematopoietic Cell Transplantation, HR = Hazard Ratio, NHB = Non-Hispanic Black, NHW = Non-Hispanic White

*Predisposing factors include age and sex.

**Enabling factors include marital status, Distressed Communities Index, region, distance to nearest transplant center, and year of diagnosis.

***Need factors include Charlson Comorbidity Index, lymphoma subtype, and Ann Arbor stage.

**Supplementary Table 9.** Cox models for stem collections for patients aged 66-74 years

| **Race/**  **Ethnicity** | **Unadjusted** | | **Adjusted for**  **Predisposing Factors*** | | **Adjusted for**  **Enabling Factors**** | | **Adjusted for**  **Need Factors***** | | **Fully Adjusted** | |
| --- | --- | --- | --- | --- | --- | --- | --- | --- | --- | --- |
|  | HR (95% CI) | *P*-value | HR (95% CI) | *P*-value | HR (95% CI) | *P*-value | HR (95% CI) | *P*-value | HR (95% CI) | *P*-value |
| NHW | Reference |  | Reference |  | Reference |  | Reference |  | Reference |  |
| NHB | 0.55 (0.32-0.96) | 0.04 | 0.54 (0.31-0.94) | 0.03 | 0.70 (0.40-1.23) | 0.21 | 0.64 (0.37-1.12) | 0.12 | 0.75 (0.43-1.32) | 0.32 |
| Asian | 0.80 (0.40-1.61) | 0.53 | 0.83 (0.41-1.67) | 0.60 | 0.79 (0.39-1.61) | 0.52 | 0.72 (0.36-1.46) | 0.36 | 0.74 (0.36-1.51) | 0.40 |
| Other | 0.50 (0.23-1.05) | 0.07 | 0.45 (0.21-0.94) | 0.04 | 0.49 (0.23-1.04) | 0.06 | 0.47 (0.22-0.98) | 0.05 | 0.44 (0.21-0.94) | 0.03 |
| Hispanic | 1.09 (0.77-1.53) | 0.63 | 1.06 (0.76-1.50) | 0.73 | 1.18 (0.83-1.68) | 0.36 | 1.09 (0.78-1.54) | 0.61 | 1.14 (0.80-1.62) | 0.48 |

Abbreviations: CI = Confidence Interval, HR = Hazard Ratio, NHB = Non-Hispanic Black, NHW = Non-Hispanic White

*Predisposing factors include age and sex.

**Enabling factors include marital status, Distressed Communities Index, region, distance to nearest transplant center, and year of diagnosis.

***Need factors include Charlson Comorbidity Index, lymphoma subtype, and Ann Arbor stage.
